# Wing variation in *Culex nigripalpus* (Diptera: Culicidae) in urban parks

**DOI:** 10.1186/s13071-017-2348-5

**Published:** 2017-09-18

**Authors:** Gabriela Cristina de Carvalho, Daniel Pagotto Vendrami, Mauro Toledo Marrelli, André Barretto Bruno Wilke

**Affiliations:** 1 Faculdade de Saúde Pública, Universidade de São Paulo, São Paulo, Brazil; 2 Instituto de Medicina Tropical, Universidade de São Paulo, São Paulo, Brazil

**Keywords:** Population structure, Wing shape variation, Urban parks, Urbanization

## Abstract

**Background:**

*Culex nigripalpus* has a wide geographical distribution and is found in North and South America. Females are considered primary vectors for several arboviruses, including Saint Louis encephalitis virus, Venezuelan equine encephalitis virus and Eastern equine encephalitis virus, as well as a potential vector of West Nile virus. In view of the epidemiological importance of this mosquito and its high abundance, this study sought to investigate wing variation in *Cx. nigripalpus* populations from urban parks in the city of São Paulo, Brazil.

**Methods:**

Female mosquitoes were collected in seven urban parks in the city of São Paulo between 2011 and 2013. Eighteen landmark coordinates from the right wing of each female mosquito were digitized, and the dissimilarities between populations were assessed by canonical variate analysis and cross-validated reclassification and by constructing a Neighbor-Joining (NJ) tree based on Mahalanobis distances. The centroid size was calculated to determine mean wing size in each population.

**Results:**

Canonical variate analysis based on fixed landmarks of the wing revealed a pattern of segregation between urban and sylvatic *Cx. nigripalpus*, a similar result to that revealed by the NJ tree topology, in which the population from Shangrilá Park segregated into a distinct branch separate from the other more urban populations.

**Conclusion:**

Environmental heterogeneity may be affecting the wing shape variation of *Cx. nigripalpus* populations*.*

**Electronic supplementary material:**

The online version of this article (10.1186/s13071-017-2348-5) contains supplementary material, which is available to authorized users.

## Background

*Culex* (*Culex*) *nigripalpus* (Theobald) is a species native to Brazil that has a tropical and subtropical distribution in North and South America. Females can lay eggs in artificial containers, thrive in urban environments and females have been reported to blood-feed on birds and humans as well as other mammals [1–8]. This feeding behavior makes the species more of a public health concern as females can vector several arboviruses, such as Saint Louis encephalitis virus (SLEV), Venezuelan equine encephalitis virus (VEEV), Eastern equine encephalitis virus (EEEV) and West Nile virus (WNV) [9–14].

The increase in anthropogenic impact and pressure on natural environments increases the risk of contact between humans and pathogens and may accelerate the spread of arthropod-borne pathogens, resulting in an increase in morbidity and mortality due to the diseases they cause, which have been identified as one of the five major emerging problems in public health [15, 16]. As many pathogens of human diseases are transmitted by mosquitoes, controlling the spread of pathogens is a major challenge [17, 18]. The growth of the human population and continuing land occupation have been dramatically modifying the landscape and changing the climate conditions, favoring the spread of vector mosquitoes capable of surviving in urbanized environments as well as an increase in their density [18–21].

In an attempt to minimize the negative effects of urbanization on the human population, urban parks have been created in large cities around the world to preserve the remaining natural habitats and create refuges for native and exotic species of fauna and flora. These parks act as “green islands” surrounded by the urban matrix [17, 22–26].

Genotypic markers are frequently used to investigate the microevolution of mosquitoes [27–31]. However, wing geometric morphometrics are not only useful for assessing microevolution events but can also provide valuable information on phenotypic variability and population structure [32–34].

We hypothesize that heterogeneous environmental areas may be correlated with the variation in shape and size of wings of *Culex nigripalpus* in populations in the city of São Paulo, Brazil*.* The objective of the present study was, therefore, to use wing geometric morphometrics to analyze wing variation patterns in *Cx. nigripalpus* females in urban parks.

## Methods

### Mosquito collections

*Culex nigripalpus* females were collected once a month for one year in seven urban parks in the city of São Paulo (except for Anhanguera Park, where collections lasted two years) using CDC light traps baited with dry ice (Table 1) [35]. All parks surveyed in this study have similar characteristics, they have been created as the city grew and there was an increase in the urban area of the city of São Paulo; also, they consist of secondary forest, except the Shangrilá Park, which is located in an adjacent area to a remnant of Atlantic Forest extending 60 km from São Paulo to the coast [36]. Mosquitoes were identified using taxonomic keys by Consoli & Lourenço de Oliveira [5].

**Table 1:** Sampling information for *Culex nigripalpus* populations collected in seven urban parks in the city of São Paulo, Brazil

Park	Coordinates	Collection year	Sample size
Anhanguera (ANG)	23°29′33.36″S, 46°45′43.50″W	2011–2013	60
Burle Marx (BMX)	23°37′55.92″S, 46°43′17.25″W	2012–2013	30
Ibirapuera (IBR)	23°35′14.40″S, 46°39′27.48″W	2011–2012	23
Piqueri (PIQ)	23°31′39.98″S, 46°34′24.98″W	2012–2013	34
Previdência (PRV)	23°34′40.99″S, 46°43′37.92″W	2012–2013	32
Santo Dias (SDS)	23°45′29.35″S, 46°46′23.18″W	2011–2012	33
Shangrilá (SHA)	23°45′29.35″S, 46°39′44.28″W	2011–2012	33

### Wing preparation and data collection

**Fig. 1:**
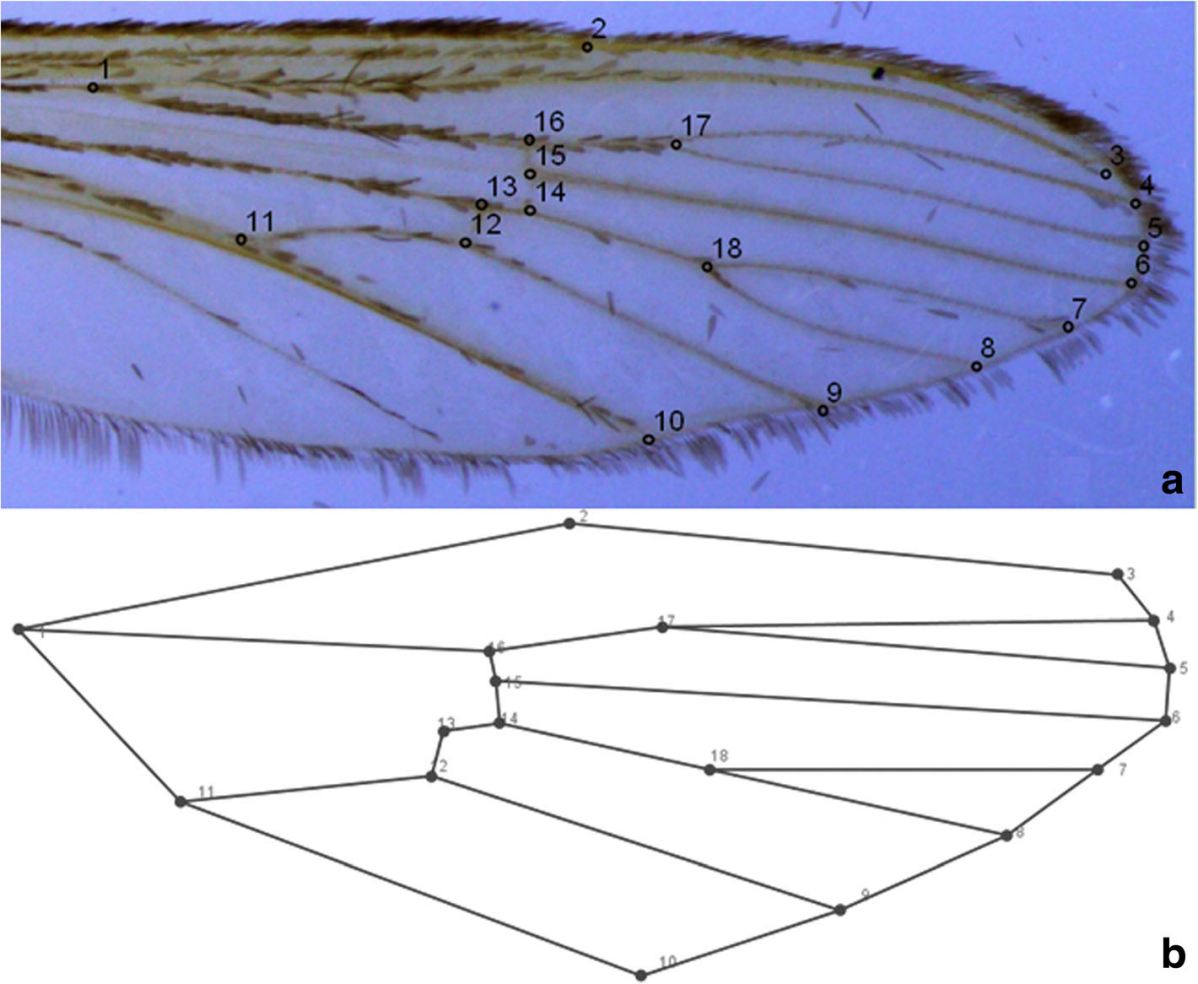
Wing of female *Culex nigripalpus*. **a** The 18 landmarks. **b** Wireframe representation of the 18 landmarks

The right wing of each female was removed from the thorax and mounted between slide and coverslip (0.08–0.12 mm) using Canada balsam (Sigma-Aldrich, St. Louis, MO, USA). The wings were then viewed using a Leica M205C stereoscope under 35× magnification. In each image, the coordinates of 18 landmarks represented by the intersections of wing veins were digitized using TpsDig software 1.4 [37] (Fig. 1).

### Morphometric approach

To assess mean wing size for each population, the isometric estimator known as centroid size (CS) [38] was calculated using MorphoJ 1.02 [39]. The results for CS were compared by non-parametric ANOVA and *post-hoc* Tukey’s test in PAST 1.89 (Table 1) [40].

The allometric effect of wing size on wing shape was estimated using multiple regression analysis of the Procrustes coordinates on CS. The statistical significance of the allometric effect was determined by non-parametric permutation testing with 10,000 randomizations in MorphoJ 1.02.

Discriminant analysis was used to explore the degree of wing shape dissimilarity between females in the morphospace produced by canonical variate analysis (CVA) using MorphoJ 1.02 and to calculate the Mahalanobis distances between samples. The latter were used to construct a NJ tree to further examine the similarities among populations with PAST 1.89. *Culex quinquefasciatus* (Say) (*n* = 30) was used as the outgroup.

The dissimilarity in wing shape between populations was estimated by cross-validated reclassification tests in MorphoJ 1.02. To test for possible isolation by distance, the correlation between Procrustes distances and geographical distances (linear kilometers) was calculated using the Mantel test in PAST 1.89.

## Results

**Fig. 2:**
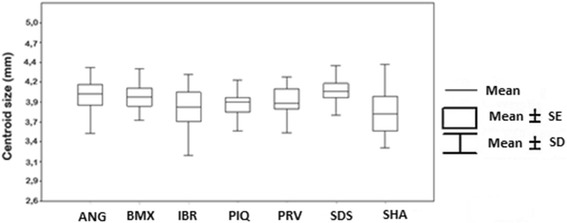
Boxplot showing mean centroid sizes of *Culex nigripalpus* wings. Differences between the following populations were statistically significant (*P* < 0.05): SDS *vs* IBR, SDS *vs* PIQ, SHA *vs* ANG, SHA *vs* BMX, and SHA *vs* SDS. *Abbreviations*: ANG, Anhanguera; BMX, Burle Marx; IBR, Ibirapuera; PIQ, Piqueri; SDS, Santo Dias; SHA, Shangrilá

Centroid size ranged from 3.25 to 4.85 mm; the SHA population had the lowest average (3.84 mm) and the SDS the highest (4.13 mm). The IBR population had the highest intra-population variation (3.25 to 4.78 mm) (Fig. 2). The statistical significance of the variation in mean CS was found between the following populations: SDS and IBR, SDS and PIQ, SHA and ANG, SHA and BMX, and SHA and SDS (ANOVA: *F*_(5,62)_ = 94.54, *P* < 0.01) (Additional file 1: Table S1).

Allometry accounted for 1.45% (*P* = 0.0027) of wing size influencing variations in wing shape and, although considered negligible, was removed from the subsequent analysis. The Mantel test showed a weak correlation between Procrustes values and geographical distance (*r* = 0.31979, *r* ^2^ = 0.10226), but this was not statistically significant (*P* = 0.1586).

**Fig. 3:**
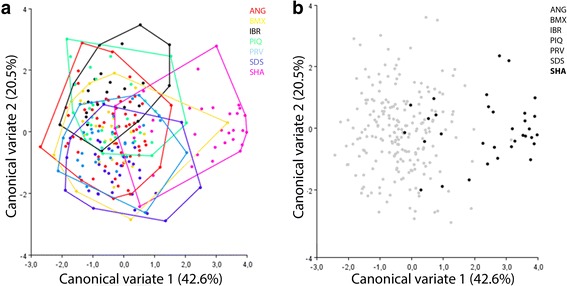
Morphological space of the first two canonical variates for *Culex nigripalpus* based on 18 wing landmarks, considering all populations (**a**); and with urban populations excluded highlighting specimens from SHA population (**b**). The relative contribution of each canonical variate is shown in parentheses

While CVA revealed a certain level of segregation between IBR, SHA and SDS, there was major overlapping between BMX, PRV, SDS, ANG, IBR and PIQ. SHA segregated into two major subpopulations, one with wing shapes similar to those of the other populations and another with wing shape patterns unlike those of any of the other populations (Fig. 3).

The SHA population was segregated in two major clusters, (i) one cluster sharing similar wing shapes with the other studied populations, labeled urban; and (ii) a second cluster composed of unique wing shape patterns, exclusively found in this subpopulation, labeled sylvatic. These labels were defined according to the characteristics of the Shangrilá Park, as a protected part of native Atlantic Forest, spreading for more than 60 km to the coast.

A cross-validated reclassification test in which the specimens were labeled urban or sylvatic yielded 96% accuracy for the urban wing shape and 100% accuracy for the sylvatic wing shape, indicating that wing shape variation was significantly different between these two groups.

**Fig. 4:**
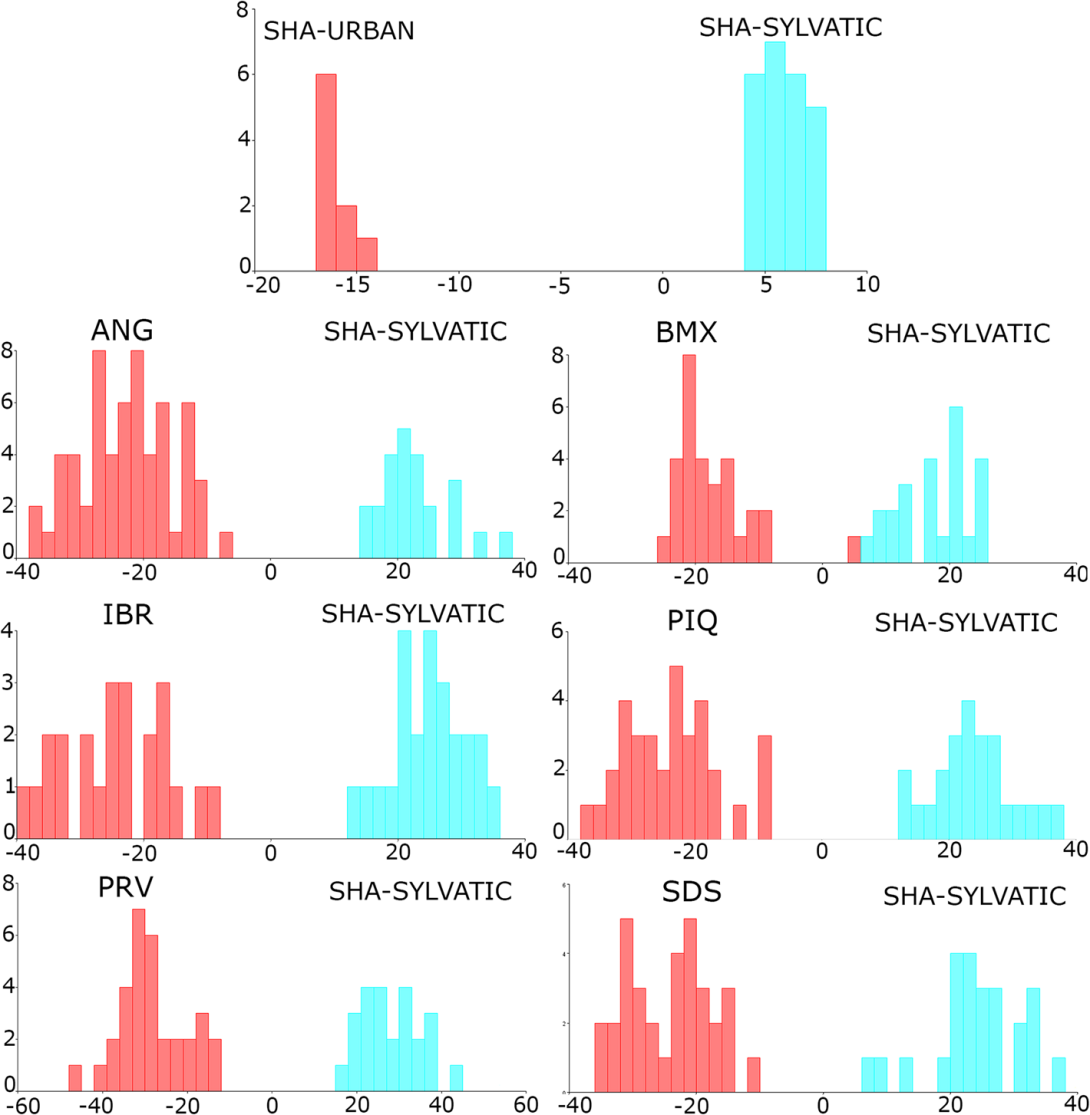
Wing shape diagram of the first canonical variable from the comparison of urban and sylvatic specimens. Blue: wing shape patterns common to sylvatic specimens found exclusively in the SHA population; red: wing shape patterns for each population. X axis: first canonical variable; Y axis: frequency. *Abbreviations*: ANG, Anhanguera; BMX, Burle Marx; IBR, Ibirapuera; PIQ, Piqueri; SDS, Santo Dias; SHA, Shangrilá

The CVA using the urban and sylvatic specimens revealed a similar pattern among the populations, but when SHA was analyzed individually, the specimens segregated into two groups, one urban (SHA-URBAN) and the other sylvatic (SHA-SYLVATIC). The sylvatic group of specimens was subsequently compared with the remaining populations, resulting in a clear pattern of segregation between the urban and sylvatic specimens for all the populations (Fig. 4). This highlights the existence of two distinct patterns of wing shapes in the populations studied.

**Fig. 5:**
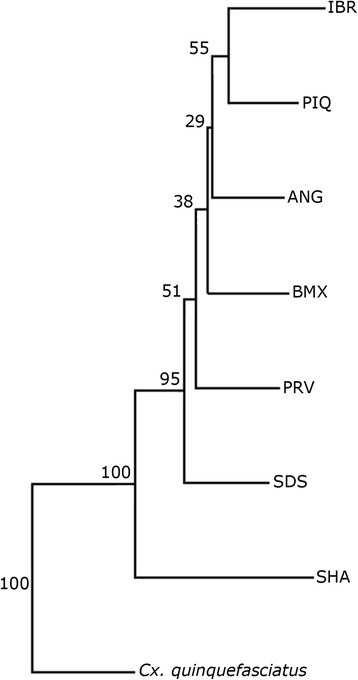
Neighbor-Joining tree for *Culex nigripalpus* based on Mahalanobis distances with 1000 bootstrap replicates. *Abbreviations*: ANG, Anhanguera; BMX, Burle Marx; IBR, Ibirapuera; PIQ, Piqueri; SDS, Santo Dias; SHA, Shangrilá

In the NJ tree, the SHA and SDS populations segregated into branches supported by high bootstrap values (100 and 95, respectively) (Fig. 5). Discriminant analysis revealed that the differences in wing shape between females of the populations were statistically significant (*P* < 0.05) except for the difference between the PIQ and IBR populations (*P* = 0.3839) (Additional file 2: Table S2).

The cross-validated reclassification tests for the SDS and IBR populations yielded a reclassification score of 80%, indicating a difference in wing shape patterns between females of these two populations. In contrast, the ANG and IBR populations had a reclassification score of 34%, indicating homogeneous wing shape patterns between females of these populations. The SHA population had the highest reclassification scores of all the populations studied, ranging from 70.5% when compared with PIQ to 88.3% when compared with ANG, indicating that females of this population have wing shape patterns that were not found in females of the other populations, increasing its reclassification scores and corroborating the CVA analysis (Table 2).

**Table 2:** Cross-validated reclassification scores (%) based on wing shape similarities for *Culex nigripalpus* populations collected in seven urban parks in the city of São Paulo, Brazil

	ANG	BMX	IBR	PIQ	PRV	SDS	SHA
ANG	–	56.6	34.7	55.8	68.7	60.6	75.7
BMX	75	–	78.2	55.8	78.1	54.5	69.6
IBR	71.6	76.6	–	52.1	60.8	65.2	69.5
PIQ	65.0	60.0	50.0	–	58.8	61.7	73.5
PRV	73.3	60.0	70.0	65.5	–	65.6	78.1
SDS	75.6	53.3	81.8	66.6	63.6	–	81.8
SHA	88.3	80.0	78.7	70.5	75.7	72.7	–

## Discussion

Our results indicate that the wing shape of *Culex nigripalpus* females is moderately heterogeneous in the populations studied. Of the seven populations analyzed, six had similar wing shapes, which can be explained by the fact that these populations were collected in areas with high levels of urbanization and anthropogenic impact, and were therefore under high selective pressures [41], resulting in lower wing shape variation. This phenomenon has been reported in previous studies of species of *Aedes* and *Anopheles* [34, 42, 43].

The SHA specimens, however, were collected in a remnant of Atlantic Forest that borders on the Billings reservoir in the city and on a very large conservation area in the Atlantic Forest. These specimens had two distinct wing shape patterns, one similar to that of the urbanized populations and another that was not found in any of the six other populations. This variation may be explained by the fact that the specimens were collected in a park containing a remnant of native Atlantic Forest, as well as the influence of urban areas near its entrance, providing two distinct habitats for *Cx. nigripalpus*. Environmental heterogeneity can affect the phenotypic patterns of organisms, driving their phenotype to local conditions.

Wing shape variability is an important trait indicator of how insects cope with environmental variations, which ultimately may affect their genomes being perceived as genetic variation at the phenotypic level. A possible explanation for the wing shape variation observed in SHA not being observed in any other population is that the vegetation of those parks was mostly complete reforestation [24]. Although it is not possible to infer that the wing shape pattern is due to specific selective pressures from the urban environment, the presence of a unique wing shape pattern found exclusively in a preserved environment may indicate a correlation between these variables. Similar wing shape variation was found for butterflies in China [44].

The urban areas are characterized as highly fragmented environments with different use and occupations, under which conditions, biological communities tend to undergo radical changes in their composition and diversity [45–47]. This phenomenon was previously seen in studies of vector-mosquitoes in the city of São Paulo, Brazil, in which urbanization was driving their population dynamics, promoting a population demographic expansion of a few species of mosquitoes that are adapted to urban areas, such as *Ae. aegypti* (Linnaeus)*, Ae. fluviatilis* (Lutz)*, Cx. quinquefasciatus* and *Cx. nigripalpus*, which are widely found throughout the urban environment [17, 31, 36, 48, 49].

The structuring of the SHA population may be related to the intraspecific variation of distinctive morphometric characters that resulted from environmental conditions from which a given population is sampled. Urbanization is known for promoting both genetic and biotic homogenization [46], consistent with the patterns seen in *Cx. nigripalpus*; similar results have been found for *Ae. aegypti* [28]. Moreover, the segregation of the SHA population into a single branch with a bootstrap value of 100 may indicate a possible retention of ancestral wing shape polymorphism, since *Cx. nigripalpus* is native to Brazil [22, 49], and Shangrilá Park is located in an urban-sylvatic transition zone, forming an ecological corridor between the Atlantic Forest and Billings Reservoir [24]. In addition, the cross-validated reclassification test indicated that females of this population have wing shape patterns different from those of the other populations studied here, corroborating the hypothesis of an exclusive wing shape pattern in the sylvatic group of specimens.

The adaptation of vector mosquitoes to urban habitats is an important selection driver that may lead to population structuring and the appearance of subpopulations. With ever-increasing urbanization, mosquitoes need to adapt to new conditions imposed by the environment, such as restricted genetic variability during the early domestication process, host-dependent dispersal, isolation and genetic drift, a favorable scenario for the emergence of subpopulations [50–54]. Furthermore, wing shape in mosquitoes is known to be a heritable trait and thus can be an indicator of evolutionary change [55]. The fact that the ANG, BMX, IBR, PIQ, PREV and SDS populations had homogeneous wing shape patterns is consistent with a genetic homogenization scenario, in which a population undergoes a demographic expansion driven by the urban environment, losing its structure in the process, as seen in a previous study of *Ae. fluviatilis* collected in similar urban parks in the city of São Paulo, Brazil [31].

The moderate wing polymorphism in the study populations detected by the CVA, NJ tree and reclassification test, and the fact that isolation by distance was not identified, indicate that urban environment has a greater influence on population structure than geographical distance in the populations studied. Similar results were found in previous studies for other mosquito species, including *Ae. aegypti* and *Ae*. *fluviatilis* [29, 31].

Population density, availability of food sources and temperature are known to modify wing size in insects [52]. The differences in centroid sizes found in our analysis probably result from the conditions in the locations where the mosquitoes originated. The SHA and SDS were the only populations that exhibited different wing sizes. Our hypothesis is that mosquitoes in the former have a high ecological valence whereas mosquitoes in the latter are found in more anthropic areas, where their larvae can develop in artificial containers. Wing variation due to habitat conditions of the immature stages has also been found in *Triatoma sordida* (Stål) and *Ae. aegypti* [56, 57].

## Conclusion

Mosquito species that can survive in urban environments tend to have an advantage over sylvatic species because of their ability to utilize different habitats for development of the immature stages and because of the easier access to hosts for blood-feeding in such environments, leading to an increase in the abundance and territorial expansion of these species [46, 58]. Therefore, the structuring pattern observed in *Cx. nigripalpus* populations, which segregated into two distinct groups (sylvatic and urban), as well as the overall low variability of wing shape resulting from high selective pressures, indicate that differences of environmental heterogeneity may have influenced wing shape in the populations studied.

## Additional files

Additional file 1: Table S1. ANOVA test for the significance of median Centroid Size differences between *Cx. nigripalpus* populations collected in seven urban parks in the city of São Paulo, Brazil. Tukey’s *post-hoc* pairwise comparisons (ANOVA: *F*_(5,62)_ = 94.54, *P* < 0.01). (DOCX 15 kb)

Additional file 2: Table S2. Values of Mahalanobis distances between *Cx. nigripalpus* populations collected in seven urban parks in the city of São Paulo, Brazil. (DOCX 14 kb)

## Abbreviations

ANG: Anhanguera
ANOVA: Analysis of variance
BMX: Burle Marx
CDC: Centers for Disease Control
CS: Centroid size
CVA: Canonical variate analysis
EEEV: Eastern equine encephalitis virus
IBR: Ibirapuera
NJ: Neighbor-joining
PIQ: Piqueri
PRV: Previdência
SDS: Santo Dias
SHA: Shangrilá
SLEV: Saint Louis encephalitis virus
VEEV: Venezuelan equine encephalitis virus
WNV: West Nile virus
