# Supplementary material for: Wing variation in Culex nigripalpus (Diptera: Culicidae) in urban parks
Source: Parasit Vectors. 2017 Sep 18;10:423. doi: 10.1186/s13071-017-2348-5 (PMC5604421; doi:10.1186/s13071-017-2348-5)
Supplement: Supplementary file 1 — ANOVA test for the significance of median Centroid Size differences between Cx. nigripalpus populations collected in seven urban parks in the city of São Paulo, Brazil. Tukey’s post-hoc pairwise comparisons (ANOVA: F (5,62) = 94.54, P < 0.01). (DOCX 15 kb) [file 13071_2017_2348_MOESM1_ESM.docx]

**Additional file 1: Table S1.** ANOVA test for the significance of median Centroid Size differences between *Cx. nigripalpus* populations collected in seven urban parks in the city of São Paulo, Brazil. Tukey's *post-hoc* pairwise comparisons (ANOVA: *F* _(5,62)_ = 94,54, *P* <0.01).

|  | Anhanguera | Burle Marx | Ibirapuera | Piqueri | Previdência | Santo Dias | Shangrilá |
| --- | --- | --- | --- | --- | --- | --- | --- |
| Anhanguera | 0 | 1 | 0.142 | 0.2685 | 0.7608 | 0.8731 | **0.004492** |
| Burle Marx | 0.1667 | 0 | 0.1853 | 0.3332 | 0.8254 | 0.8175 | **0.006948** |
| Ibirapuera | 3.605 | 3.439 | 0 | 0.9999 | 0.9352 | **0.002741** | 0.9211 |
| Piqueri | 3.183 | 3.016 | 0.4225 | 0 | 0.9871 | **0.008355** | 0.7899 |
| Previdência | 2.084 | 1.917 | 1.522 | 1.099 | 0 | 0.09174 | 0.2955 |
| Santo Dias | 1.772 | 1.939 | 5.377 | 4.955 | 3.856 | 0 | **<0.0001** |
| Shangrilá | 5.195 | 5.028 | 1.589 | 2.012 | 3.111 | 6.967 | 0 |

Pairwise ANOVA comparisons values (below diagonal)*. P* values (above diagonal). Significant values (*P*<0.01) in bold.
