# Supplementary material for: Wing variation in Culex nigripalpus (Diptera: Culicidae) in urban parks
Source: Parasit Vectors. 2017 Sep 18;10:423. doi: 10.1186/s13071-017-2348-5 (PMC5604421; doi:10.1186/s13071-017-2348-5)
Supplement: Supplementary file 2 — Values of Mahalanobis distances between Cx. nigripalpus populations collected in seven urban parks in the city of São Paulo, Brazil. (DOCX 14 kb) [file 13071_2017_2348_MOESM2_ESM.docx]

**Additional file 2: Table S2.** Values of Mahalanobis distances between *Cx. nigripalpus* populations collected in seven urban parks in the city of São Paulo, Brazil.

|  | Anhanguera | Burle Marx | Ibirapuera | Piqueri | Previdência | Santo Dias | Shangrilá |
| --- | --- | --- | --- | --- | --- | --- | --- |
| Anhanguera | 0 | **0..0033** | **0..0005** | **0.0165** | **<.0001** | **<.0001** | **<.0001** |
| Burle Marx | 1.6282 | 0 | **0.0443** | **0.0049** | **0.0002** | **0.0013** | **<.0001** |
| Ibirapuera | 1.9803 | 2.0202 | 0 | 0.3839 | **0.0008** | **<.0001** | **<.0001** |
| Piqueri | 1.478 | 1.8354 | 1.6989 | 0 | **0.002** | **<.0001** | **<.0001** |
| Previdência | 1.8624 | 1.922 | 2.1872 | 1.778 | 0 | **0.0021** | **<.0001** |
| Santo Dias | 1.8088 | 1.9207 | 2.5802 | 2.0987 | 1.7741 | 0 | **<.0001** |
| Shangrilá | 2.8565 | 3.0659 | 3.2112 | 2.9521 | 3.1257 | 2.8615 | 0 |

Mahalanobis distance values (below diagonal). *P* values (above diagonal). Significant values (*P*<0.05) in bold.
